# Increased Access to Immunoglobulin Replacement Therapy for Patients with Primary Immunodeficiency in Poland Based on Clinical Usage Data of Immunoglobulin G over a 5-Year Period

**DOI:** 10.3390/jcm12062431

**Published:** 2023-03-22

**Authors:** Ewa Więsik-Szewczyk, Marcin Ziętkiewicz, Anna Radziwilska-Muc, Karina Jahnz-Różyk

**Affiliations:** 1 Department of Internal Medicine, Pneumonology, Allergology and Clinical Immunology, Central Clinical Hospital of the Ministry of National Defense, Military Institute of Medicine, 04-349 Warsaw, Poland; 2 Department of Rheumatology, Clinical Immunology, Geriatrics and Internal Medicine, Medical University of Gdańsk, 80-214 Gdańsk, Poland

**Keywords:** immunoglobulins G indications, subcutaneous immunoglobulin replacement therapy, off-label indications, clinical practice, rational use, real-world data

## Abstract

Owing to the rising popularity and demand for immunoglobulins (IgG), obtaining supplies and rationalizing IgG use have become challenging. Herein, IgG consumption in Poland was analyzed through total IgG use and number of patients reported to the National Health Fund between 1 January 2016 and 31 December 2020. Total IgG used within 5 years increased by 27.48%, IgG use/1000 inhabitants/year was 23.13 g (2016) and 29.61 g (2020). In 2020, 35.5 % of IgG used was for neurological conditions, 25% for primary immunodeficiencies (PID), and 39.3% for all other indications. Within 5 years, 1,121,168.75 g IgG was used in PID; the use increased by 72%, from 783 in 2016 to 1153 patients in 2020. The proportion of patients who received subcutaneous immunoglobulin (SCIG) replacement therapy (IgRT) increased to 78% (2020). Within 5 years, 1,783,534.81 g IgG was used in neurological drug programs (+42.44%) and 2,327,513.88 g (+1.25%) outside neurological indications and outside PID. The annual IgG amount decreased in adult anesthesiology and intensive care (−46%), internal medicine (−55%), pneumonology (−50%), pediatric clinical immunology (−50%), and gynecology and obstetrics (−48%) and increased in dermatology (+178%), rheumatology (+103%), and clinical transplantation (+82%). IgG use significantly increased in Poland, mostly owing to PID. Subcutaneous IgG administration is currently the most common mode of IgRT in PID patients. An increase in SCIG administration may be expected for other indications. Implementing evidence-based clinical guidelines is key to prioritizing and rationalizing IgG use for immunomodulatory indications and secondary immune deficiencies.

## 1. Introduction

Polyvalent immunoglobulin G (IgG) preparations are obtained by the fractionation of human plasma from multiple donors. Most preparations contain over 95% IgG and trace amounts of immunoglobulin M (IgM) and immunoglobulin A (IgA). Currently, the indications for IgG treatment have broadened, although not all of them are supported by satisfactory evidence-based data. An increase in the demand and consumption of IgG has been reported worldwide. The annual growth rate of global immunoglobulin use, based on data from the Marketing Research Bureau, reflected an average year on year rise in immunoglobulin use between 2010 and 2018 of approximately 12% per year [1]. This occurs simultaneously with decreased plasma donation. Product shortages and supply chain disruptions increased during the COVID-19 pandemic, when increased demand and a drop in plasma and blood donations coincided. Since “the product follows the price”, there have been difficulties in obtaining supplies in countries with lower acquisition prices [2]. This is the case in various European countries, including Poland [2,3]. This has inspired the search for solutions to increase plasma donations and to critically analyze clinical practice for the therapeutic use of IgG. Indications for IgG therapy and reimbursement policies vary significantly among countries and regions, particularly for off-label use. IgG replacement therapy (IgRT) for patients with primary immunodeficiencies (PID) is an example of a treatment that is long-term and life-saving, as well as evidence-based. The use of immunosuppressive IgG in Kawasaki disease is an example of the immunomodulatory properties of IgG [4]. Up to 50% of IgG consumption is for conditions for which there are no absolute indications or for which alternative therapies might be used, such as targeted biological therapies for inflammatory rheumatologic diseases or thrombopoietin agonists for immune thrombocytopenia [5,6]. IgG preparations were based on the WHO List of Essential Medicines [7]. They are unique biological preparations, with no single product or method of administration that is suitable for all patients. Knowledge of the actual IgG consumption allows the optimization of drug management and ensures treatment continuity in patients for whom it is a vital indication, even in times of crisis and inaccessibility to plasma-derived products. To the best of our knowledge, such a comprehensive analysis has never been conducted in Poland. To fill this gap, we analyzed the evolution of IgG consumption in Poland. We collected real-world longitudinal data for reported IgG applications for different indications in the national public health care system.

### Reimbursement of IgG in Poland

In Poland, innovative treatments with expensive active ingredients have been reimbursed by the National Health Fund in special drug programs. In drug programs, the treatment is administered for select diseases and includes a strictly defined group of patients. The content of each drug program is published as an annex to the notice of the Minister of Health on the list of the Reimbursement of Drugs, Food Products for Special Dietary Purposes, and Medical Devices [8]. The description of the program included patient eligibility, exclusion and inclusion criteria, drug regimen, method of administration, a list of diagnostic tests performed to determine the patient’s eligibility, and the necessity to monitor and assess the efficacy of treatment. Patients eligible for the drug programs were treated free of charge. 

IgG treatment in PID and in select neurological conditions is reimbursed by dedicated drug programs. The drug program, which covers the treatment of children with PID, was introduced to the Polish health system in 2000 [9], followed by a drug program for adults introduced in 2015. As part of these programs, patients with the following diagnoses received IgRT: common variable immunodeficiency (CVID), agammaglobulinemia, unspecified primary hypogammaglobulinemia, syndromic immunodeficiencies with hypogammaglobulinemia, significant subclass deficiency, and specific antibody deficiency. Indications coded according to ICD-10 are presented in the supplementary materials (Table S1). Immunoglobulins are administered either intravenously (IVIG) or subcutaneously (SCIG). IVIG must be administered in a hospital or one-day care unit. Two methods of SCIG application are currently available: conventional (either using an infusion pump or without, termed rapid-push SCIG) and facilitated (fSCIG), which are aided by the initial administration of human recombinant hyaluronidase in the same needle as IgG. The subcutaneous preparations can be self-administered at home. Immunoglobulin subcutaneous home therapy must be initiated in the hospital or in an outpatient unit where the patient is educated on the principles of self-home treatment.

A drug program in which IgG treatment was reimbursement to patients with select neurological conditions was introduced to the Polish health system in 2015. As part of this program, patients with the following diseases were treated with IgG: chronic inflammatory demyelinating polyneuropathy (CIDP), multifocal motor neuropathy (MMN), myasthenia gravis, paraneoplastic syndromes including Lambert–Eaton myasthenic syndrome, inflammation of the limbic system, motor or motor sensory polyneuropathy, Guillain–Barre syndrome, idiopathic inflammatory myositis (polymyositis and dermatomyositis), neuromyelitis optica, and encephalitis with antibodies to neuronal antigens. During the analysis period, IgG for neurological conditions was administered intravenously in the hospital. Table S1 in the supplementary materials includes a list of neurological indications coded according to the ICD-10.

IgG treatments are reimbursed in other indications, including labeled indications, such as idiopathic thrombocytopenic purpura (ITP), secondary immunodeficiencies (SID), and Kawasaki diseases, as well as off-label indications, if administered in a unit that has a contract with the National Health Fund. No additional approval of the reimbursement policy was required. The assessment of indication is based on the competence of the treating physician. In contrast to drug programs, patient eligibility, monitoring, and efficacy assessments are not strictly regulated.

## 2. Materials and Methods

### 2.1. Data Collection

We analyzed the total IgG use reported to the National Health Fund database between 1 January 2016 and 31 December 2020. Data on the annual number of patients and overall consumption of IgG were recorded. Due to reimbursement regulations and National Health Fund reporting rules for IgG treatment, data were collected in three main subsets: (1) primary immune deficiency drug programs (PID DPs), separately for children and adults; (2) neurological drug program of immunoglobulin treatment; and (3) other indications codified with ICD-10. We identified the proportion of total IgG consumption among the different medical disciplines. The reports provided in the database did not include individual patient data. We obtained aggregate data on IgG consumption and number of patients treated each year for a specified indication. We calculated the average values for each year for each patient by dividing the number of grams of IgG administered for a given indication by the number of patients treated.

For all data analysis, authors received permission from the National Health Fund.

### 2.2. Ethic Statement

The reports provided in the database did not include individual patient data. The separate approval for this study was not needed.

## 3. Results

### 3.1. Total IgG Consumption

**Figure 1:**
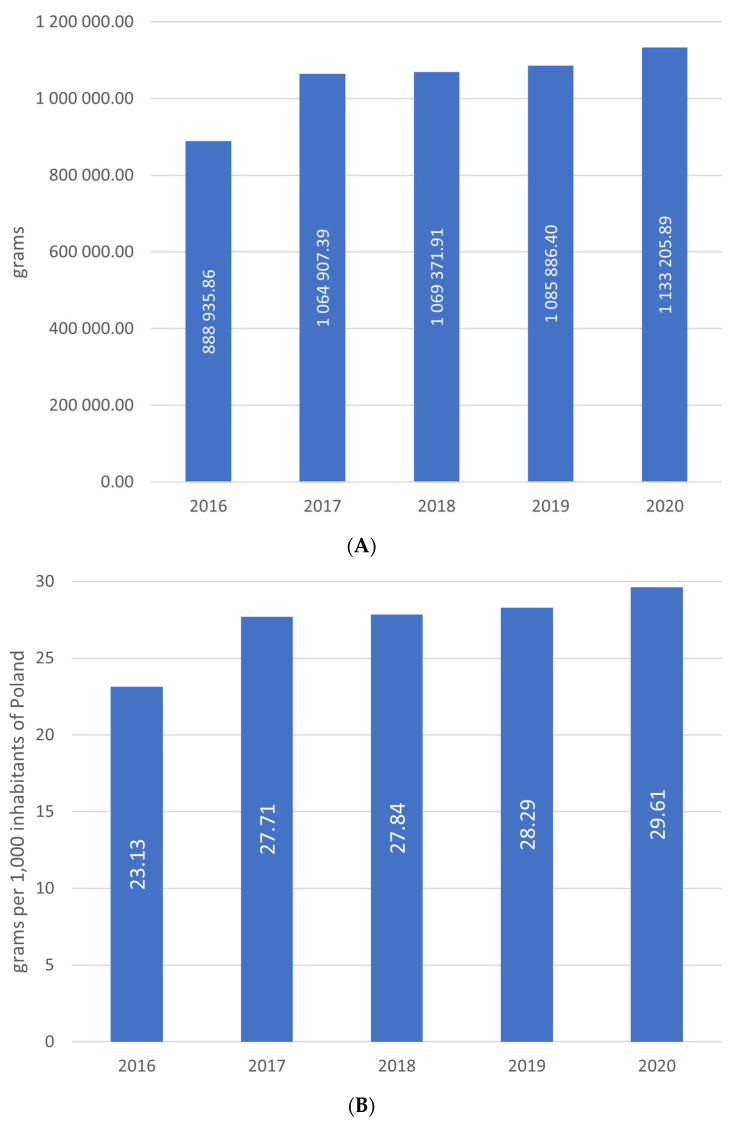

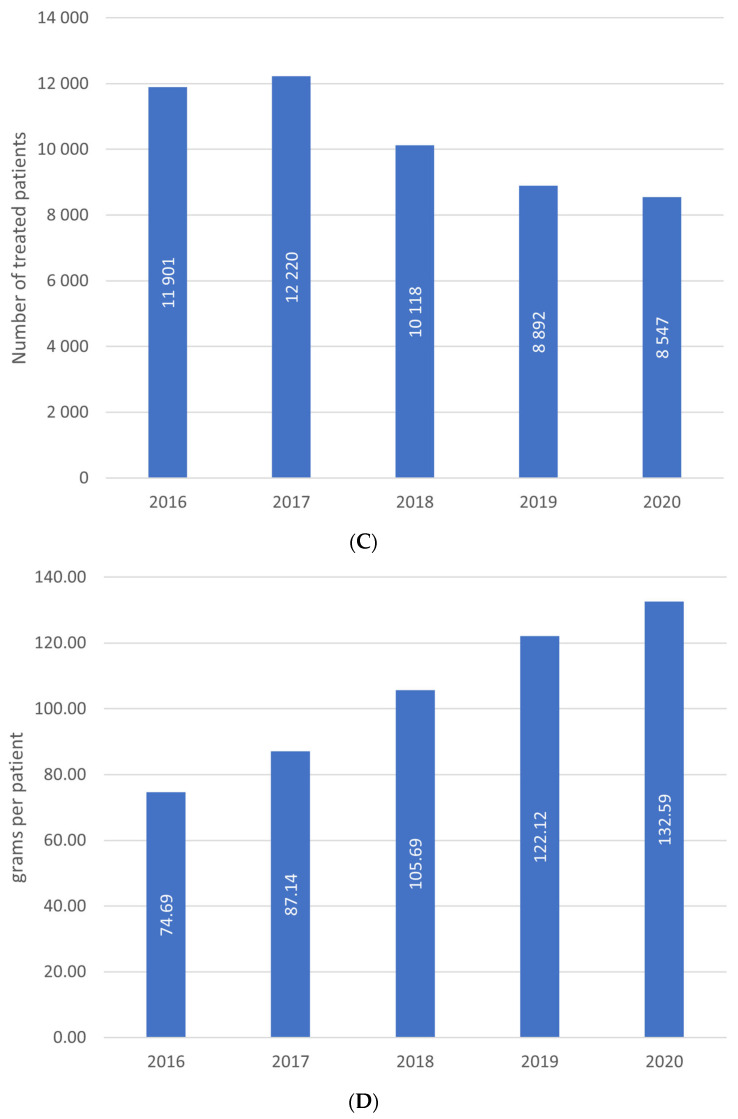
(**A**) Total annual consumption of immunoglobulins (in grams) in Poland. (**B**) Total annual consumption of immunoglobulins per 1000 inhabitants in Poland. (**C**) Total number of patients treated of immunoglobulins per year in Poland. (**D**) Mean annual consumption of immunoglobulins in Poland.

The total amount of IgG consumed in Poland between 2016 and 2020 was 5,232,217.44 g. The annual amount used increased from 888,935.85 g in 2016 to 1,133,205.89 g in 2020 (+244,270 g, +27.48%) (Figure 1A). The calculated annual amount per 1000 inhabitants was 23.13 g in 2016 and 29.61 g in 2020 (Figure 1B). The number of treated patients annually ranged from the highest, 12,220, 2017 to the lowest, 8547, in 2020 (Figure 1C). During the 5-year observation period, the mean annual IgG consumption per patient was 101.25 g and increased from 74.69 g in 2016 to 132.9 g in 2020 (Figure 1D).

### 3.2. IgG Consumption in Primary Immune Deficiencies Drug Programs

**Figure 2:**
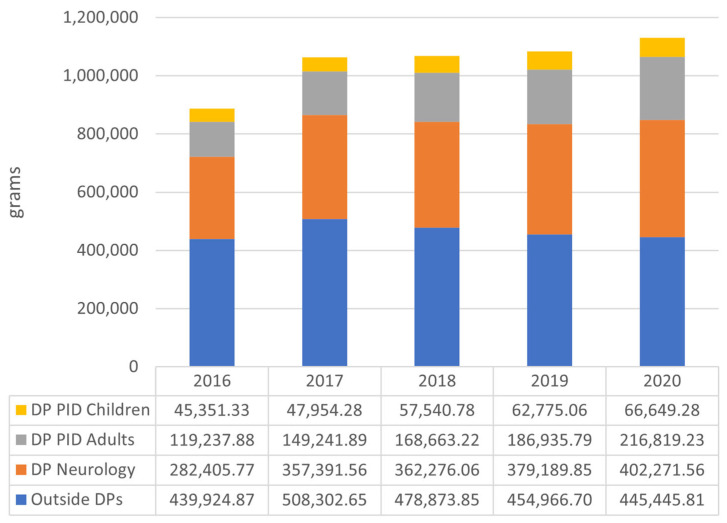
Immunoglobulin use (in grams) reported annually in Poland divided into drug programs and other indications. DP: drug program; PID: primary immunodeficiency. DPs: drug programs.

Within 5 years, IgG administered to PID patients (adults and children) accounted for 21.4% of total IgG (1,121,168.75 g). The annual IgG share in PID patients increased from 18.5% in 2016 to 25% in 2020 (Figure 2). 

**Figure 3:**
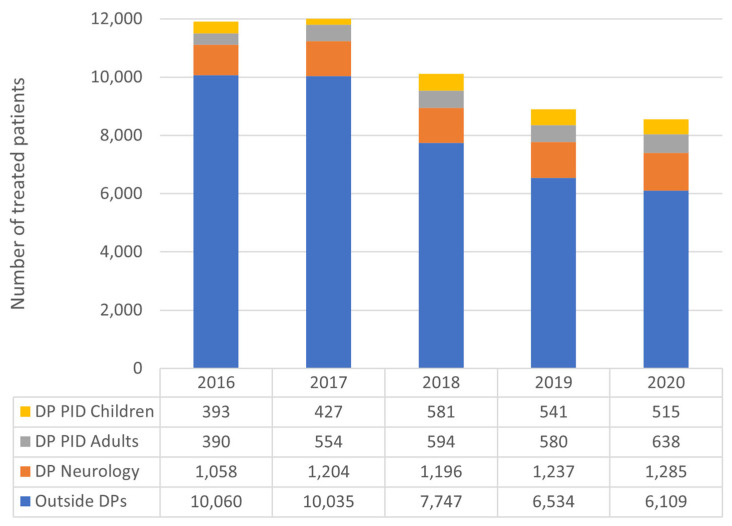
The number of patients treated annually with immunoglobulins, divided into drug programs and other indications. DP: drug program. PID: primary immunodeficiency. DPs: drug programs.

The amount of IgG annually used in PID increased from 164,589.21 g to 283,468.52 g (+118,879.31, +72.23%). The number of treated PID patients was 783 in 2016 and 1153 in 2020 (Figure 3). 

**Figure 4:**
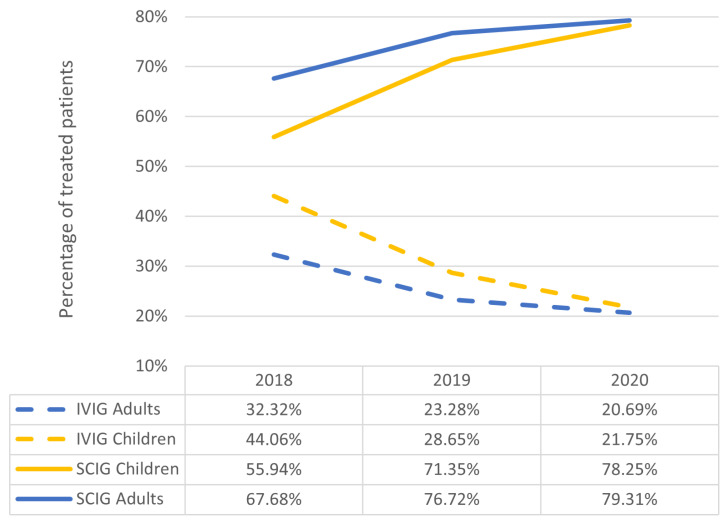
The proportion of patients treated through SCIG and IVIG in primary deficiency drug programs in adults and children.

The most significant increase in the number of treated patients was observed in adults. The number of adult patients treated per year changed from 390 to 554, between 2016 and 2017. Subsequently, it gradually increased to 638 adult patients with PID in 2020 (Figure 3). The mean consumption per patient for 5 years was between 115 g (2016) and 129 g (2020) in children and between 305 g (2016) and 339 g (2020) in adults. PID patients have been administered IgG through IVIG and SCIG, including fSCIG, since 2016. The annual use of SCIG was 85,731.24 g in 2016 and 238,385.13 g in 2020 (+152,653.73 g, +178.06%). In 2020, 506 of 638 adult patients (79%) and 402 of 515 children (78%) received SCIG (Figure 4).

### 3.3. IgG Consumption in Neurological Drug Program

The neurological drug program accounted for 34.1% of total IgG consumption over a 5-year period. Annual share was 31.8% in 2016 and 35.5% in 2020. Annual IgG consumption in the neurology drug program increased from 282,405.77 g in 2016 to 402,271.56 g in 2020 (+119,865.79 g, +42.44%) (Figure 2). The number of treated patients increased from 1058 to 1285 (Figure 3). The mean annual consumption per patient for five years was between 266 in 2016 and 313 g in 2020.

### 3.4. IgG Consumption in Indications Outside Drug Programs

The third analyzed subset included all reported IgG use outside the drug programs, selected neurological conditions and PID, within the National Health Fund. The IgG usage in this subset accounted for 44.5% of overall 5-year IgG consumption. Annual share of overall IgG use decreased from 49.5% in 2016 to 39.3% in 2020. Annual amount of IgG use increased from 439,924.87 g in 2016 to 445,445.81 g in 2020 (+5520.93 g, +1.25%) (Figure 2). The number of treated patients decreased from 10,060 to 6109 (Figure 3). The consumption per patient for five years was between 43.72 g in 2016 and 72.92 g in 2020.

Outside drug programs, the most IgG was used in hematology, pediatric oncology and hematology, clinical immunology, pediatrics, rheumatology, neurology, anesthesiology, and intensive care (Table 1 and Table 2).

There were different trends among specialties over the 5-year period studied. 

The annual IgG consumption decreased in adult anesthesiology and intensive care (25,145.50 g to 13,578 g, −46%), internal medicine (19,369.5 g to 8775 g, −55%), pneumonology (4692.2 g to 2332 g, −50%), pediatric clinical immunology (4503 g to 2001 g, −56%), and gynecology and obstetrics (17,168 g to 8961.86 g, −48%). 

The annual consumption of IgG increased in dermatology (7141 g to 19,882 g, +178%), rheumatology (15,337 g to 31,129.5 g, +103%), and clinical transplantation (2351 g to 4269 g, +82%). The results of changes in annual IgG consumption in different specialties are presented in Table 1. 

Outside the drug programs, we analyzed the most common indications codified according to ICD-10 from three perspectives: total amount of IgG used within 5 years (Table S2), amount of IgG used per patient per year (Table S2), and number of treated patients (Table S3). Within 5 years, the highest total amount of IgG was used for the following indications (Table S2): purpura and other hematologic conditions (D69), lymphoid leukemia (C91), other immunodeficiencies (D84), encounter with other aftercare and medical care (Z51), and other polyneuropathies (G62).

The following top five indications were reported according to the number of treated patients (Table S3): purpura and other hematologic conditions (D69), lymphoid leukemia (C91), other immunodeficiencies (D84), encounter with full-term uncomplicated delivery (O80), and encounter with other aftercare and medical care (Z51). The number of patients reported with secondary immunodeficiencies (SID), coded as D84, C91, C92, and C83, was 1502 in 2020.

The highest amount of annual IgG per patient was used for the following indications: pemphigus (L10), other polyneuropathies (G62), dermatomyositis (M33), systemic lupus erythematosus (M32), other necrotizing vasculopathies (M31).

Specific data for the top indications and the amount of IgG used outside PID and neurological drug programs are presented in the supplementary materials (Tables S2 and S3).

## 4. Discussion

This is the first comprehensive population-based assessment to examine real-world IgG use over five years in Poland. We documented a global increase in IgG use of 27.48% in Poland, which is higher than the average reported among the top countries (+9–12%) [10]. This may be related to the low baseline IgG consumption in 2016. We demonstrated that the consumption of IgG was 29.61 g/1000 inhabitants in 2020. In terms of population, the consumption of IgG (g/1000 inhabitants) differs worldwide. The highest consumption has been reported in the United States (210 g, 2017), Australia (206 g, 2015), and Canada (179 g, 2015). In Europe [3], available data ranged from 115 g in Germany (2018) to 33.1 g in the Czech Republic (2014) [2,3,7,11]. The annual number of treated patients decreased; however, it was accompanied by an increase in the total volume of IgG administered per patient, which is in line with results of another publication [6]. Moreover, there were different trends in IgG use depending on medical discipline. We found that the increased use of IgG in PID had the most significant impact on IgG consumption in Poland. The number of treated PID patients increased from 673 in 2014 to 1153 in 2020 [9]. The most significant increase was observed in adults, whose number almost tripled since 2014 (233 vs. 638) [9]. This is the result of increased awareness of PID, improved diagnostic tools, and reduction in diagnostic delay [12]. The milestone was the introduction of a drug program for adult patients with PID in 2015. This can explain the rapid increase in the number of treated patients between 2016 and 2017. Drug programs in PID cover the availability of all modes of IgG administration, including home SCIG treatment, which is one of the principles of care in PID [13]. We have shown that the majority of PID patients (78%) have received SCIG since 2014, when SCIG therapy was less often used (26% of adults and 35% of children) according to report from the Polish Immunodeficiency Working Group [9]. The proportion of adults was reversed in 2016, and the demand for SCIG accelerated when fSCIG became widely available [14,15]. The choice of mode of treatment is based on shared patient and medical team decisions [15]; however, life-long SCIG treatment may be beneficial from an economic perspective [16,17,18,19,20]. An example is a cost minimization model to evaluate costs of IVIG versus SCIG from the Spanish National Healthcare System perspective, with the assumption that all IVIG infusions were administered in a hospital and 95% of SCIG infusions were administered at home. This model is similar to our system and suggests that SCIG may be a cost-saving alternative to IVIG in patients with PID [21].

In our analysis, patients in neurological drug programs remained most common users of IgG (35% in 2020) for indications with adequate evidence support [22]. These results are in agreement with other published data and latent immunoglobulin demand models [1,2,23]. Within the period of our analysis, only IVIG was administered for neurological indications. However, SCIG products have also been approved for CIPD. If available, SCIG home therapy is often preferred by patients. Moreover, there are possible economic benefits for the Health Care System [24]. Even though the daily cost of SCIG in the initial phase of CIDP treatment was higher than hospital-based IVIG in terms of health insurance, the additional costs were compensated during the maintenance phase (from week 28) [17]. Keeping this in mind, we can estimate that SCIG product approval for treatment of neurological conditions significantly increases the demand for SCIG, which might impact its availability. 

The data described in the third subset of our study reflected the patterns of IgG use in different medical disciplines and indications, including off-label use. This analysis illustrates the clinical practice in Poland in an area where most Polish hospitals do not have specific guidelines for IgG use and reimbursement, in contrast to drug programs. From the perspective of IgG amount and number of treated patients, hematologic indications dominated, including immune thrombocytopenia and SID. However, compared to PID, the number of patients with SID was lower than expected. Data from the Australian National Blood Authority showed the highest amount of secondary antibody deficiency in hematological malignancies or hematopoietic stem cell transplantation (HSCT), which is more than twice that of primary immunodeficiency (PID) [23]. Similar results were obtained in the UK [1] where SID appears to be increasing the demand for IgGRT. Recommendations for SID treatment differ worldwide, and in Poland, they are not strictly described [25]. Moreover, home SCIG therapy is not reimbursed for patients with SID in Poland. In our opinion, this is an area that needs improvement. The implementation of a registry and audit of compliance would help assess the needs and tools for treatment efficacy in patients with SID [26]. In addition to drug programs, immunoglobulins were used for weak, off-label indications and indications where the benefit of IgG treatment is doubtful or unclear. These were reported in addition to other medical care and injuries (Z51 and T94) and encounters for full-term uncomplicated delivery (O80). Therefore, IgG could potentially be overused for indications that are not well supported by recent evidence. We were able to demonstrate that consumption in specialties, adult anesthesiology and intensive care, gynecology and obstetrics, and infectious diseases, related to some of these indicators decreased, which is in line with current recommendations. Finally, we found that the annual consumption of IgG was highly increased in dermatology and rheumatology fields. There are some potential areas of reduction in IgG use for immunomodulatory indications because new targeted therapies are now available for pemphigus, pemphigoid, SLE, and vasculitis.

Our study has some limitations. The National Health Fund database does not include individual patient data. We aggregated the data on IgG consumption and the number of patients treated each year for a specified indication. We could not precisely define the indications reported outside the drug programs. This methodology is not reproducible because the system of reporting data is specific to the Polish Health Fund. However, this is the first comprehensive summary of IgG use in Poland based on real-world data. We have described the reimbursement policy to make the data comparable to the results from other countries. To date, no national registry for PID has been established in Poland. Therefore, our data represent the real-world number of patients treated for primary antibody deficiencies in Poland.

## 5. Conclusions

We demonstrated a dynamic increase in IgG use in Poland, with a growing impact on the treatment of patients with PID. The subcutaneous route of IgG administration is currently the most common mode of IgRT in PID patients. Furthermore, we need to improve IgRT in patients with SID. Future studies should benefit from a comparative assessment of IVIG and SCIG administration. Evidence-based clinical guidelines need to be implemented to prioritize and rationalize IgG use for immunomodulatory indications. Periodical reports should be spread among different specialties involved in IgG treatment to update the clinical revision of IgG efficacy, availability, and possible treatment alternatives.

## Figures and Tables

**Table 1:** Annual consumption of immunoglobulins in grams, total consumption from 2016 to 2020, and change in consumption over this period (2020 vs. 2016) by specialty. NR not reported.

SPECIALTY	2016	2017	2018	2019	2020	TOTAL AMOUNT	CHANGE 2016 vs. 2020
HEMATOLOGY	183,776.60	222,100.00	195,407.00	178,641.75	162,866.00	942,791.35	−11.38%
PEDIATRIC ONCOHEMATOLOGY	36,619.00	42,090.35	44,060.45	32,015.50	31,870.50	186,655.80	−12.97%
CLINICAL IMMUNOLOGY	22,875.85	27,698.55	29,515.15	31,456.65	42,223.50	153,769.70	84.58%
PEDIATRICS	26,672.00	28,055.50	25,518.00	28,044.00	34,471.00	142,760.50	29.24%
REUMATOLOGY	15,337.00	27,770.50	21,864.00	27,871.00	31,129.50	123,972.00	102.97%
NEUROLOGY	22,552.00	20,574.00	26,527.00	27,448.00	21,571.00	118,672.00	−4.35%
ANESTHESIOLOGY AND INTENSIVE THERAPY	25,145.50	23,485.00	25,138.50	20,509.00	13,578.00	107,856.00	−46.00%
ONCOLOGY	16,502.40	19,339.00	19,342.00	16,154.00	13,551.00	84,888.40	−17.88%
INTERNAL MEDICINE	19,369.50	18,313.00	19,517.00	15,781.00	8775.00	81,755.50	−54.70%
OBSTETRICS AND GYNECOLOGY	17,168.00	17,534.00	8872.00	9582.00	8961.86	62,117.86	−47.80%
DERMATOLOGY AND VENEROLOGY	7141.00	8712.00	9974.00	15,254.00	19,882.00	60,963.00	178.42%
PEDIATRIC NEUROLOGY	6687.00	8852.00	9011.50	12,597.50	13,927.00	51,075.00	108.27%
ALERGOLOGY	6609.50	7520.00	5984.00	6721.00	7590.00	34,424.50	14.83%
NEFROLOGY	5741.00	7835.00	8978.00	5410.00	5954.00	33,918.00	3.71%
PEDIATRIC ANESTHESIOLOGY AND INTENSIVE THERAPY	5018.90	4057.55	4441.25	4650.70	5979.95	24,148.35	19.15%
PEDIATRIC REUMATOLOGY	3131.50	4502.00	4224.00	4595.00	4732.50	21,185.00	51.13%
LUNG DISEASES	4692.20	4113.00	4897.20	4312.00	2332.00	20,346.40	−50.30%
PEDIATRIC CLINICAL IMMUNOLOGY	4503.00	3547.20	3672.80	2806.60	2001.00	16,530.60	−55.56%
TRANSPLANTOLOGY	2351.00	1694.00	3042.00	3246.00	4269.00	14,602.00	81.58%
PEDIATRIC INFECTIOUS DISEASES	1911.50	2826.50	2259.00	2600.50	3305.50	12,903.00	72.93%
PEDIATRIC CARDILOGY	1233.50	1412.00	1085.00	1117.50	1991.50	6839.50	61.45%
PEDIATRIC ALLERGOLOGY	1638.00	2018.50	976.50	340.00	495.00	5468.00	−69.78%
INFECTIOUS DISEASES	788.20	1498.00	1249.00	1228.00	200.00	4963.20	−74.63%
PEDIATRIC TRANSPLANTOLOGY	564.50	725.00	1269.50	1082.50	895.00	4536.50	58.55%
PEDIATRIC LUNG DISEASES	786.70	432.50	542.50	1054.50	1218.50	4034.70	54.89%
PEDIATRIC NEFROLOGY	974.52	942.50	729.50	274.00	457.50	3378.02	−53.05%
CARDIOLOGY	120.00	605.00	727.00	174.00	309.00	1935.00	157.50%
GERIATRICS	15.00	50.00	50.00	NR	865.00	980.00	5666.67%
CARDIAC SURGERY	NR	NR	NR	NR	44.00	44.00	

**Table 2:** Mean annual consumption of immunoglobulins in grams per patient from 2016 to 2020 and mean annual dose per patient over this period by specialty. NR—not reported.

SPECIALTY	2016	2017	2018	2019	2020	Mean Over 5-Years
HEMATOLOGY	102.27	98.84	96.40	105.58	95.52	99.58
PEDIATRIC ONCOHEMATOLOGY	48.37	51.96	51.53	38.62	43.96	46.95
CLINICAL IMMUNOLOGY	97.34	109.91	119.49	116.94	150.80	119.85
PEDIATRICS	38.49	44.04	35.64	41.18	45.42	40.95
REUMATOLOGY	125.71	169.33	155.06	207.99	213.22	175.35
NEUROLOGY	145.50	162.00	158.84	180.58	163.42	161.90
ANESTHESIOLOGY AND INTENSIVE THERAPY	60.01	61.16	59.85	63.69	64.05	61.39
ONCOLOGY	63.96	50.89	56.72	60.28	69.14	58.83
INTERNAL MEDICINE	84.58	84.00	79.66	76.61	79.77	81.11
OBSTETRICS AND GYNECOLOGY	5.14	5.25	8.56	15.58	14.64	6.94
DERMATOLOGY AND VENEROLOGY	285.64	290.40	343.93	526.00	473.38	393.31
PEDIATRIC NEUROLOGY	79.61	85.12	72.67	91.29	94.10	85.41
ALERGOLOGY	101.68	115.69	213.71	231.76	210.83	154.37
NEFROLOGY	55.74	66.97	72.40	77.29	106.32	72.17
PEDIATRIC ANESTHESIOLOGY AND INTENSIVE THERAPY	11.13	10.19	9.74	10.82	17.43	11.62
PEDIATRIC REUMATOLOGY	54.94	91.88	100.57	135.15	115.43	95.00
LUNG DISEASES	111.72	117.51	106.46	100.28	86.37	105.42
PEDIATRIC CLINICAL IMMUNOLOGY	45.48	45.48	48.33	43.85	50.03	46.30
TRANSPLANTOLOGY	31.77	30.80	26.22	36.89	40.27	33.26
PEDIATRIC INFECTIOUS DISEASES	15.93	25.93	28.24	26.54	45.28	26.88
PEDIATRIC CARDILOGY	18.98	25.21	22.60	26.61	31.12	24.87
PEDIATRIC ALLERGOLOGY	40.95	36.04	17.44	17.89	24.75	28.63
INFECTIOUS DISEASES	20.74	42.80	32.87	30.70	40.00	31.82
PEDIATRIC TRANSPLANTOLOGY	22.58	31.52	45.34	36.08	44.75	36.00
PEDIATRIC LUNG DISEASES	13.11	8.16	9.86	15.51	21.76	13.82
PEDIATRIC NEFROLOGY	12.34	17.45	15.20	10.54	16.94	14.44
CARDIOLOGY	30.00	55.00	90.88	43.50	38.63	55.29
GERIATRICS	15.00	50.00	50.00	NR	216.25	140.00
CARDIAC SURGERY	NR	NR	NR	NR	7.33	7.33

## Data Availability

Full source data available on request from authors.

## Supplementary Materials

The following supporting information can be downloaded at: https://www.mdpi.com/article/10.3390/jcm12062431/s1, Table S1: Indications for immunoglobulin G treatments in drug programs codified by ICD-10; Table S2: Immunoglobulin use in grams and grams per patient reported annually in Poland in 20 most common indications outside primary immune deficiency drug program and neurology drug program reported with ICD-10 codes; Table S3: The number of patients treated annually with immunoglobulins in Poland in 20 most common indications outside primary immune deficiency drug program and neurology drug program, reported with ICD-10 codes.
