# Supplementary material for: Increased Access to Immunoglobulin Replacement Therapy for Patients with Primary Immunodeficiency in Poland Based on Clinical Usage Data of Immunoglobulin G over a 5-Year Period
Source: J Clin Med. 2023 Mar 22;12(6):2431. doi: 10.3390/jcm12062431 (PMC10059916; doi:10.3390/jcm12062431)
Supplement: Supplementary file 1 [file jcm-12-02431-s001.zip › jcm-2225840-supplementary.pdf]

Supplementary Materials:

Table S1. Indications for immunoglobulin G treatments in drug programs codified by ICD-10.

Primary Immune Deficiency drug program
D.80.0 Hereditary hipogammaglobulinaemia
D.80.1 Non-family hipogammaglobulinaemia
D.80.3 Selective deficiency of immunoglobulin G subclasses (IgG)
D.80.4 Immunodeficiency with increased levels of IgM
D.80.5 Deficiency of serum immunoglobulin antibodies similar to normal or hypergammaglobulinemia
D.80.8 Other immunodeficiencies with defect prevalence of antibodies
D.80.9 Unspecified immunodeficiency with predominant antibody defect
D.81.9 Determined combined immunodeficiency
D.82.0 Wiskott Aldrich syndrome
D.82.1 Di George syndrome
D.82.3 Deficiency response to infection with EB virus
D.82.8 Deficiency associated with other serious defects
D.82.9 Indefinite immunodeficiency associated with severe defects
D.83.0 Common variable immunodeficiency with a predominance of dysfunction or the number of B cells
D.83.1 Common variable immunodeficiency disorders predominantly related immunoregulatory T cells
D.83.8 Other common immunodeficiency
D.83.9 Indefinite common immunodeficiency
D.89.9 Determined disorders involving the immune mechanism
**Neurology drug program**
G61.8 Other inflammatory polyneuropathies
G62.8 *Other specified polyneuropathies*
G63.1 *Polyneuropathy in neoplastic disease*
G70, Myasthenia *gravis and other myoneural disorders*.
G04.8 *Other encephalitis, myelitis and encephalomyelitis*.
G73.1 *Lambert-Eaton syndrome in neoplastic disease*
G73.2, Other myasthenic syndromes in neoplastic disease
G72.4 Inflammatory and immune myopathies, not elsewhere classified
G61.0 Guillain-Barre syndrome
G36.0 Neuromyelitis optica [Devic]
M33.0 Juvenile dermatomyositis
M33.1 Other dermatomyositis
M33.2 Polymyositis.

Table S2. Immunoglobulin use in grams and grams per patient reported annually in Poland in 20 most common indications outside primary immune deficiency drug program and neurology drug program reported with ICD-10 codes.

ICD 10 CODE	2016	2017	2018	2019	2020	TOTAL
D69 PURPURA AND OTHER HAEMORRHAGIC CONDITIONS	108,664.00 (124.47)	128,904.25 (137.72)	130,543.00 (127.86)	111,756.66 (133.2)	90,477.12 (135.24)	570,345.03 (131.48)
C91 LYMPHOID LEUKAEMIA	54,513.30 (68.4)	70,213.50 (80.34)	60,851.90 (68.22)	55,328.48 (72.32)	50,909.70 (70.51)	291,816.88 (72.05)
D84 OTHER IMMUNODEFICIENCIES	50,355.90 (87.27)	57,552.35 (91.35)	59,346.65 (89.24)	58,960.74 (93.59)	62,315.47 (110.68)	288,531.11 (94.14)
Z51 OTHER MEDICAL CARE	29,296.50 (41.56)	30,445.50 (43.93)	20,289.55 (37.02)	16,854.93 (40.23)	18,362.40 (41.45)	115,248.88 (41.04)
G62 OTHER POLYNEUROPATHIES	8,405.00 (182.72)	6,963.50 (232.12)	8,407.50 (191.08)	10,817.56 (208.03)	6,778.25 (188.28)	41,371.81 (198.9)
C92 MYELOID LEUKAEMIA	7,359.50 (41.82)	7,472.20 (44.74)	7,999.50 (47.62)	7,337.24 (52.79)	9,950.10 (59.94)	40,118.54 (49.16)
J96 RESPIRATORY FAILURE	9,507.90 (42.64)	6,383.80 (43.43)	7,402.40 (40.67)	9,835.11 (48.93)	6,334.03 (42.51)	39,463.24 (43.75)

C85 OTHER AND UNSPECIFIED TYPES OF NON-HODGKIN LYMPHOMA	6,101.00 (48.81)	8,174.00 (61.92)	7,306.50 (58.45)	9,310.98 (64.66)	7,551.25 (50.34)	38,443.73 (56.87)
M32 SYSTEMIC LUPUS ERYTHEMATOSUS	4,822.00 (92.73)	8,239.00 (158.44)	6,296.00 (121.08)	8,107.21 (150.13)	10,633.80 (204.5)	38,098.01 (145.41)
M31 OTHER NECROTIZING VASCULOPATHIES	4,182.50 (95.06)	8,952.00 (129.74)	8,278.00 (131.4)	9,502.20 (190.04)	7,136.00 (148.67)	38,050.70 (138.87)
M33 DERMATOPOLYMYOSITIS	3,425.00 (142.71)	6,729.50 (177.09)	6,773.00 (218.48)	7,463.40 (213.24)	8,086.00 (224.61)	32,476.90 (198.03)
O99 OTHER MATERNAL DISEASES CLASSIFIABLE ELSEWHERE BUT COMPLICATING PREGNANCY, CHILDBIRTH AND THE PUERPERIUM	7,534.00 (99.13)	11,489.00 (113.75)	3,957.00 (197.85)	6,098.55 (184.8)	3,296.16 (193.89)	32,374.71 (131.07)
M30 POLYARTERITIS NODOSA AND RELATED CONDITIONS	5,048.00 (34.11)	5,605.00 (37.37)	5,023.00 (35.37)	4,956.95 (34.66)	10,334.34 (45.13)	30,967.29 (38.14)
C90 MULTIPLE MYELOMA AND MALIGNANT PLASMA CELL NEOPLASMS	6,459.00 (48.20)	7,653.00 (46.66)	5,129.00 (39.76)	5,108.24 (38.41)	6,138.20 (44.80)	30,487.44 (43.74)
G61 INFLAMMATORY POLYNEUROPATHY	6,468.50 (122.05)	5,304.00 (115.3)	6,374.50 (120.27)	6,260.75 (118.13)	5,920.68 (137.69)	30,328.43 (122.29)
A41 OTHER SEPSIS	4,366.50 (22.86)	6,090.00 (33.65)	4,589.50 (27.48)	3,786.31 (27.24)	7,414.88 (45.49)	26,247.19 (31.21)
D47 OTHER NEOPLASMS OF UNCERTAIN OR UNKNOWN BEHAVIOUR OF LYMPHOID, HAEMATOPOIETIC AND RELATED TISSUE	3,362.00 (101.88)	4,983.50 (113.26)	5,644.50 (112.89)	3,569.50 (67.35)	5,663.50 (117.99)	23,223.00 (101.86)
L10 PEMPHIGUS	2,749.00 (458.17)	4,596.00 (510.67)	3,049.00 (338.78)	3,619.00 (517.00)	6,790.00 (617.27)	20,803.00 (495.31)
D46 MYELODYSPLASTIC SYNDROMES	3,105.50 (83.93)	5,116.00 (89.75)	3,852.00 (87.55)	3,899.45 (77.99)	4,623.30 (90.65)	20,596.25 (86.18)
G04 ENCEPHALITIS, MYELITIS AND ENCEPHALOMYELITIS	1,596.00 (53.20)	3,240.50 (81.01)	4,565.50 (108.70)	4,841.61 (79.37)	5,576.91 (118.66)	19,820.52 (90.09)

Table S3. The number of patients treated annually with immunoglobulins in Poland in 20 most common indications outside primary immune deficiency drug program and neurology drug program, reported with ICD-10 codes.

**YEAR** / **ICD 10 CODE**	**2016**	**2017**	**2018**	**2019**	**2020**	**Total over 5-years**
D69 PURPURA AND OTHER HAEMORRHAGIC CONDITIONS	873	936	1,021	839	669	4,338
C91 LYMPHOID LEUKAEMIA	797	874	892	765	722	4,050
D84 OTHER IMMUNODEFICIENCIES	577	630	665	630	563	3,065
080 SINGLE SPONTANEOUS DELIVERY	1,131,	1,149,	370	179	167	2,996
Z51 OTHER MEDICAL CARE	705	693	548	419	443	2,808
O82 SINGLE DELIVERY BY CAESAREAN SECTION	1,027	982	235	112	137	2,493
J96 RESPIRATORY FAILURE	223	147	182	201	149	902
A41 OTHER SEPSIS	191	181	167	139	163	841
C92 MYELOID LEUKAEMIA	176	167	168	139	166	816
M30 POLYARTERITIS NODOSA AND RELATED CONDITIONS	148	150	142	143	229	812
O02 OTHER ABNORMAL PRODUCTS OF CONCEPTION	232	281	102	74	59	748
C90 MULTIPLE MYELOMA AND MALIGNANT PLASMA CELL NEOPLASMS	134	164	129	133	137	697
C85 OTHER AND UNSPECIFIED TYPES OF NON-HODGKIN LYMPHOMA	125	132	125	144	150	676
O03 FETUS AND NEWBORN AFFECTED BY OTHER COMPLICATIONS OF LABOUR AND DELIVERY	150	147	97	82	65	541
J18 PNEUMONIA, ORGANISM UNSPECIFIED	90	71	81	44	32	318
C83 NON-FOLLICULAR LYMPHOMA	74	72	61	50	51	308

J15 BACTERIAL PNEUMONIA, NOT ELSEWHERE CLAS-SIFIED	73	70	60	59	32	294
Z84 TRANSPLANTED ORGAN AND TISSUE STATUS	41	34	39	79	100	293
M31 OTHER NECROTIZING VASCULOPATHIES	44	69	63	50	48	274
P28 OMPHALITIS OF NEWBORN	46	46	36	80	59	267
